# The association of gene rearrangement and lymphoma diagnosis: A prospective observational study

**DOI:** 10.1097/MD.0000000000020733

**Published:** 2020-06-12

**Authors:** Xiaoyan He, Pei Xu, Xianwei Wang, Shuming Jiang, Daoyin Gong, Ning Wu

**Affiliations:** a Department of Pathology, Deyang People's Hospital, Deyang City; b Department of Pathology, Affiliated Hospital of Chengdu University of Traditional Chinese Medicine, Chengdu, Sichuan Province, China.

**Keywords:** gene rearrangement, lymphoma, mutation, T cell receptor

## Abstract

**Introduction:**

To investigate the gene rearrangement and mutation of lymphoma biomarkers including (Immunoglobulin H (IgH), Immunoglobulin kappa (IGK), Immunoglobulin lambda (IGL), and TCR) in the lymphoma diagnosis.

**Methods and analysis:**

Paraffin tissue samples from 240 cases diagnosed as suspected lymphoma in the department of pathology, Deyang City People's Hospital from June 2020 to June 2021 will be enrolled. Deoxyribonucleic acid extraction and Polymerase Chain Reaction (PCR) amplification will be performed in these paraffin tissue samples. Immunoglobulin and T cell receptor (TCR) rearrangement will be analyzed by hetero-double chain gel electrophoresis and BioMed-2 standardized immunoglobulin gene rearrangement detection system. In this study protocol IGH gene rearrangement, IGK gene rearrangement, both IGH and IGL gene rearrangement, both IGH and IGK gene rearrangement, both IGK and IGL gene rearrangement, both IGH, IGK and IGL gene rearrangement, TCR gene rearrangement and positive Ig/TCR rearrangement will be analyzed.

**Discussion:**

In this study, we will use B and T cell lymphoma analysis focusing on IgH, IGK, IGL, and TCR gene rearrangement, so as to provide early guidance for the diagnosis of lymphoma. Second generation sequencing technology is helpful in the differential diagnosis of lymphoma.

**Trial registration:**

Chinese Clinical trial registry: ChiCTR2000032366.

## 1 Introduction

Hematologic malignancies are divided into 5 categories: Hodgkin lymphoma, non-Hodgkin lymphoma (NHL), myeloma and acute and chronic leukemia.^[1]^ There are many types of NHL, meanwhile, diffuse large b-cell lymphoma (DLBCL) is the most common type in adults.^[2]^ Approximately one third of DLBCL patients cannot be cured by standard immunochemotherapy due to the high heterogeneity and multiple factors (such as age and gender).^[3,4]^ Therefore, different therapeutic approaches are needed, for instance, morphological, genetic, immunophenotypic and clinical tools.^[5]^

Genome rearrangement is an important oncogenic mechanism for human tumors. Detection of immunoglobulin (IG) and T cell receptor (TCR) gene rearrangement may be specific markers for lymphocyte cloning and hence indicators of lymphoma onset.^[6]^ Gene mutation detect is a new and useful approach for diagnosis of malignant lymphocyte cloning when combining histopathology and immunophenotypic analysis.^[7]^ Recently, biomed-2 cloning analysis technology has solved the problem of false positive results caused by traditional Polymerase Chain Reaction (PCR) and has gradually become a recognized standard for PCR-based Ig/TCR cloning detection.^[8–10]^ This study protocol aims to survey the clinical value of gene rearrangement and mutation in lymphoma diagnosis.

## 2 Materials and methods

### 2.1 Main aims

We aim to clarify the association of gene rearrangement and lymphoma diagnosis.

### 2.2 Study registration

The protocol scheme matches PRISMA's reporting standards. This study protocol was registered on Chinese Clinical trial registry (http://www.chictr.org.cn/index.aspx) with an ID of ChiCTR2000032366.

### 2.3 Participants

Paraffin tissue samples from 240 cases diagnosed as suspected lymphoma in the department of pathology, Deyang City People's Hospital from June 2020 to June 2021 will be collected.

#### 2.3.1 Inclusion criteria

Tissue samples of Patients who are suspected to be lymphoma, regardless of lymphoma types age, sex, inside or outside the lymph nodes, will be included.

#### 2.3.2 Exclusion criteria

Basic clinical information of patients is not complete.

The size of paraffin tissue could not meet the test requirements of HE, immunohistochemistry, and gene rearrangement will be excluded.

#### 2.3.3 Diagnostic criteria

2008 WHO classification criteria for hematopoietic and lymphoid tumors.

### 2.4 Data collection

#### 2.4.1 HE and immunohistochemical detection

Paraffin embedded tissues will be sectioned with a thickness of 4 μm, and HE staining will be carried out by automatic HE staining apparatus. Immunohistochemical staining will be performed with DAKO Link48 automatic immunohistochemical staining apparatus. The selected immunohistochemical markers include CD20, CD3, CD79, CD5, CD4, CD8, TIA-1, GranzymeB, CD56, CD10, MUM1, Bcl-2, Bcl-6, CyclinD1, CD30, and CD15.

#### 2.4.2 Deoxyribonucleic acid (DNA) extraction and rearrangement detection

Genomic Deoxyribonucleic acid (DNA) will be extracted from 240 paraffin embedding tissue samples. The IGH, Immunoglobulin kappa (IGK), LGL, T cell receptor gamma (TCRG), T cell receptor delta (TCRD), and T cell receptor beta (TCRB) rearrangement in genomic DNA will be analyzed using the European biomed-2 system. Additionally, the Next-generation sequencing (NGS) technology will be adopted for probing mutations. The detected mutations in lymphoma associated genes will be recorded, as well as the mutation ratio (the percentage of reads of this mutant site to the total number of reads that cover this site).

### 2.5 Statistical plan

Excel will be used to establish the database, and SPSS 22.0 statistical software will be used for statistical analysis in this study.

### 2.6 Dissemination

This study has been approved by the ethics committee of Deyang people's hospital. All participants immediate family members will sign the informed consent after being informed about the goals and methods of the study. The present study will be conducted in accordance with Declaration of Helsinki.

## 3 Discussion

There are 2 categories of lymphoma: Hodgkin lymphomas and NHL.^[11,12]^ NHL can be driven by genetic and environmental risk factors.^[13]^ DLBCL is the most common adult lymphoid malignancies^[14,15]^ which is consistent with our analysis. It is known that DLBCL is more common in adults than in children, with a dramatic increase in incidence at 50 years old.^[16]^ Although some patients can be relieved after treatment, due to the non-specific molecular pathogenesis of DLBCL^[17]^ and its significant heterogeneity, some advanced-stage patients cannot be cured by standard immunochemotherapy.^[12]^ Therefore, early detection and treatment of DLBCL are still a challenging problem. To date, gene analysis combined with histopathology and immunophenotypic analysis can provide a new approach for the diagnosis of malignant tumors.^[18]^ Genomic rearrangement is an important oncogenic mechanism in human tumors,^[19]^ which may produce fusion transcripts encoding chimeric proteins with new functions.^[6]^ Many studies have shown that the IG gene rearrangement is a specific marker of B lymphocyte cloning^[19,20]^ and can be used in diagnosis of B cell lymphoma.^[21,22]^ The detection of IG gene rearrangement can help determine the nature of the lesion and distinguish between benign or malignant lymphocytes.^[23,24]^ Additionally, TCR rearrangement is another criterion for the diagnosis of lymphocyte disease.^[10,25]^ Based on the PCR of IG and TCR, it has been standardized in the suspicious lymph node hyperplasia.^[26,27]^ However, early PCR strategy always induce false negatives and false positives, and it is unable to accurately distinguish between monoclonal and polyclonal PCR products.^[28,29]^ When taking the advantages of standardized BioMed-2, the disadvantages of traditional PCR assay can be addressed,^[8,30]^ and this multiple detection showed a great clinical value for PCR based Ig/TCR cloning detection.^[31]^

In conclusion, standardization of BioMed-2 gene rearrangement detection system is a powerful tool of lymphoma diagnosis, IGH, IGK, Immunoglobulin lambda (IGL) and TCR gene rearrangement distributions are distinct in different subtypes of lymphoma.

## Author contributions

Xiaoyan He and Ning Wu conceived the idea for this study; Pei Xu provided statistical plan; Xiaoyan and Xianwei Wang drafted the protocol. Daoyin Gong, Shuming Jiang and Ning Wu reviewed the protocol and provided critical feedback. All authors approved the article in its final form.
